# Simultaneous brain and lung metastases of pancreatic ductal adenocarcinoma after curative pancreatectomy: a case report and literature review

**DOI:** 10.1186/s12876-020-01587-3

**Published:** 2021-01-06

**Authors:** Yoshito Oka, Shigetsugu Takano, Yusuke Kouchi, Katsunori Furukawa, Tsukasa Takayashiki, Satoshi Kuboki, Daisuke Suzuki, Nozomu Sakai, Shingo Kagawa, Isamu Hosokawa, Takashi Mishima, Takanori Konishi, Takashi Kishimoto, Masayuki Ohtsuka

**Affiliations:** 1 Department of General Surgery, Chiba University Graduate School of Medicine, 1-8-1 Inohana, Chuo-ku, Chiba, 260-8677 Japan; 2 Department of Molecular Pathology, Chiba University, Chuo-ku, Chiba, 260-8677 Japan

**Keywords:** Pancreatic ductal adenocarcinoma, Brain metastasis, Lung metastasis, Carbonic anhydrase IX

## Abstract

**Background:**

Pancreatic ductal adenocarcinoma (PDAC) rarely metastasizes to the brain; therefore, the features of brain metastasis of PDAC are still unknown. We encountered simultaneous metastases to the brain and lung in a PDAC patient after curative surgery.

Case presentation

A 68-year-old man with PDAC in the tail of the pancreas underwent distal pancreato-splenectomy. He received gemcitabine as adjuvant chemotherapy for 6 months. Two months later, brain and lung metastases occurred simultaneously. Considering the systemic condition, the patient received gamma knife treatment and an Ommaya reservoir was inserted for drainage. The patient’s condition gradually worsened and he received the best supportive care. To the best of our knowledge, only 28 cases in which brain metastases of PDAC were identified at the time of ante-mortem have been reported to date, including the present case. Notably, the percentage of simultaneous brain and lung metastases was higher (32%) in a series of reviewed cohorts. Thus, lung metastasis might be one of the risk factors for the development of brain metastasis in patients with PDAC. As a systemic disease, it can be inferred that neoplastic cells will develop brain metastasis via hematogenous dissemination beyond the blood–brain barrier, even if local recurrence is controlled. In our case, immunohistochemical staining showed that the neoplastic cells were positive for carbonic anhydrase 9 (CAIX), mucin core protein 1 (MUC1), and MUC5AC in the resected primary PDAC.

**Conclusion:**

We describe a case of simultaneous brain and lung metastases of PDAC after curative pancreatectomy, review previous literature, and discuss the clinical features of brain metastasis of PDAC.

## Background

Pancreatic ductal adenocarcinoma (PDAC) is a fatal disease with a 5-year survival rate of 9% [1]. With curative resection, the median survival time for patients is only 23–36 months [2]. Because of the absence of early signs or symptoms of PDAC, 65–70% of all the patients show nodal or distant metastases to the liver, peritoneum, lungs or bone by the time of diagnosis [3]. Brain metastases from PDAC are extremely rare, occurring in 0.57% of all metastases of PDAC [4], and survival ranges from 2 weeks to more than 10 years (Table 1). Although several cases have been reported, the majority of such cases have been identified postmortem [5]. This is due to the fact that most patients do not survive long enough to experience the clinical manifestations of brain metastases. It is also possible that brain imaging studies are not routinely performed in patients with cancer who have no neurological symptoms. Herein, we report a case of simultaneous brain and lung metastases of PDAC after curative pancreatectomy and summarize previous reports of brain metastases of PDAC.

## Case presentation

**Fig. 1:**
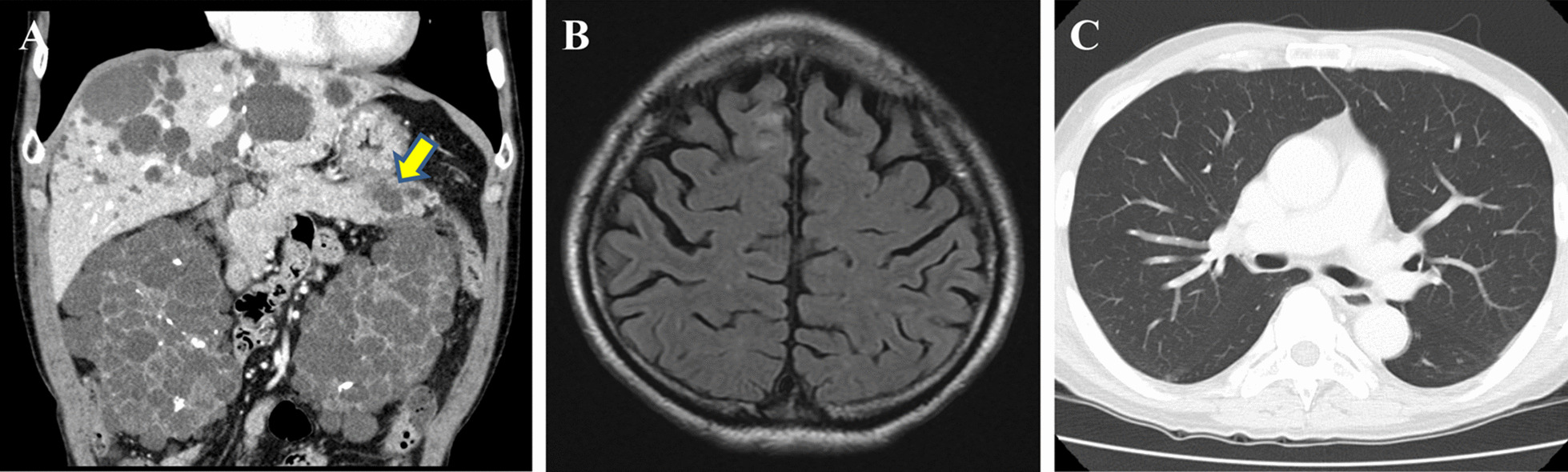
Radiological images at the time of diagnosis. **a** Computed tomography revealed a low-density mass in the tail of the pancreas (yellow arrow), and polycystic liver and kidney. **b** No metastases were observed in the lungs. **c** MRI image revealed no metastasis in the brain

**Fig. 2:**
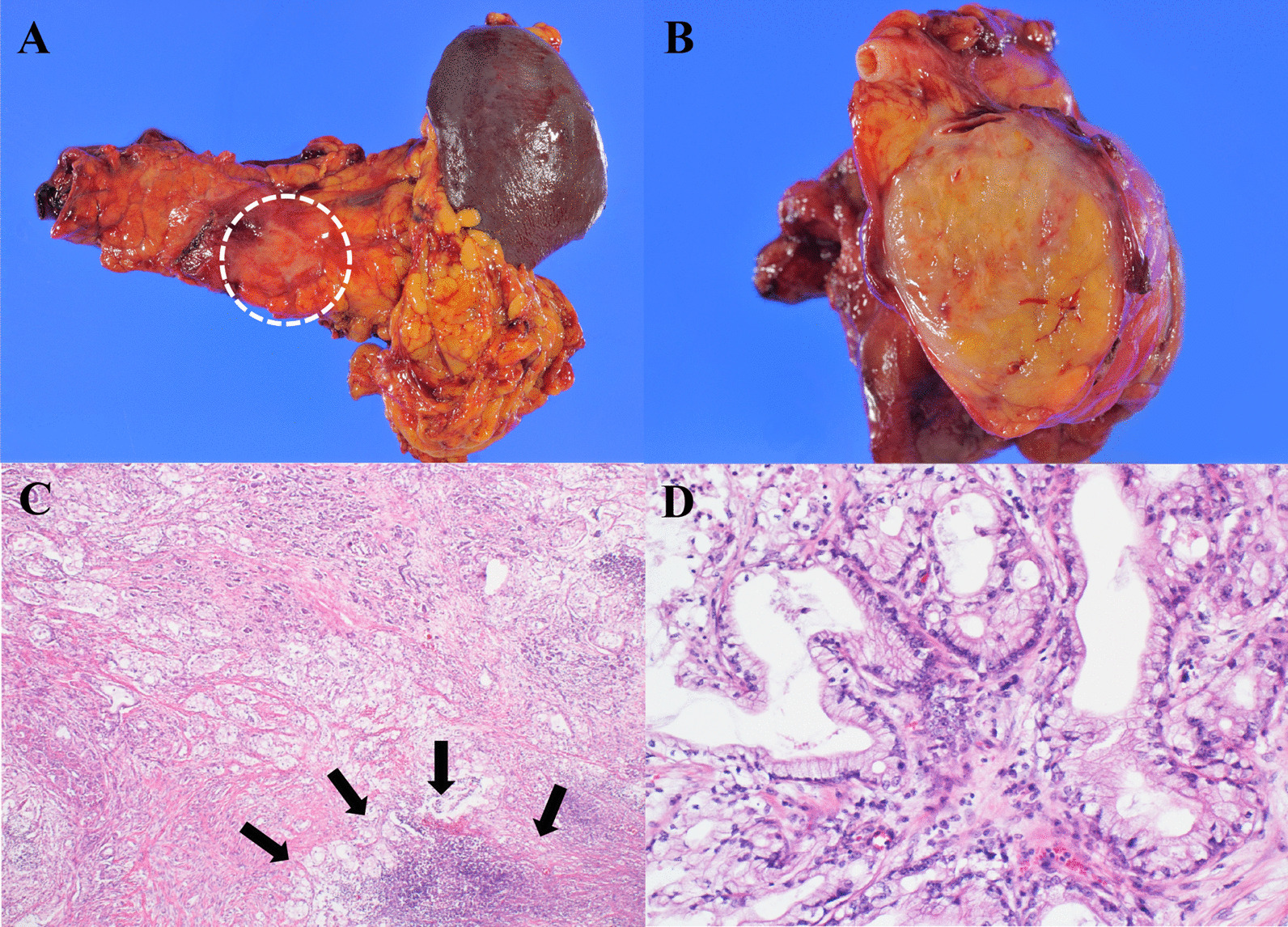
Macroscopic and histopathological findings. **a**, **b** Macroscopic images of the resected pancreatic tumor showed a white to tan solid mass in the pancreatic tail (white dashed circle). **c** Histologically, neoplastic cells infiltrate forming small to medium sized glands, and in some area, luminal formation becomes inconspicuous. Necrosis are also obvious in the neoplastic tissue (black arrows). **d** The neoplastic cells are mainly composed of columnar cells with clear foamy cytoplasm and apical brush border-like zone. Staining with HE. Original magnification: × 40 (C) and × 200 (D)

**Fig. 3:**
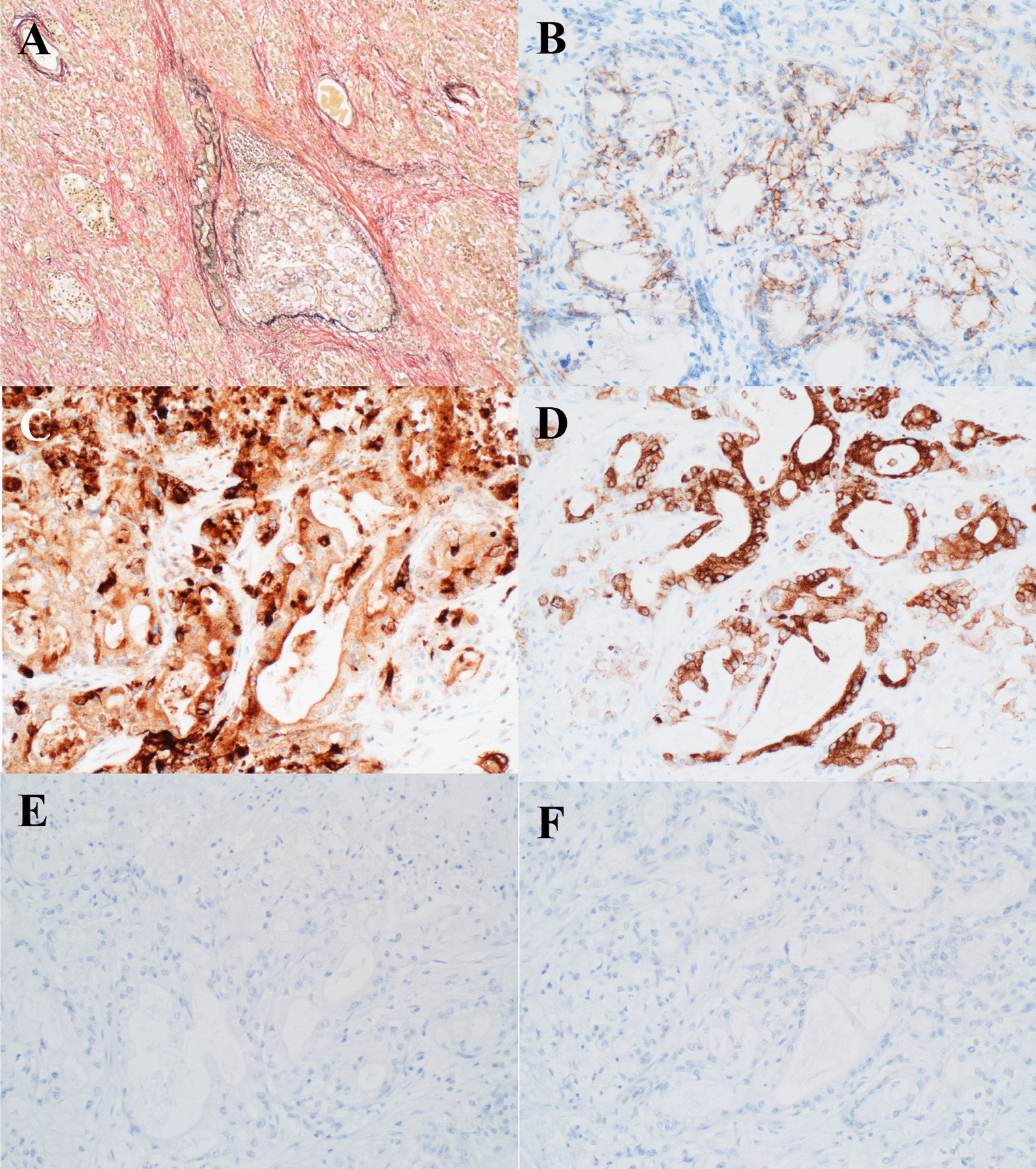
Histochemical findings and Immunochemical features of the resected primary PDAC. **a** Microscopic finding of venous infiltration by Elastica van Gieson staining. **b** CAIX is expressed on the cellular membrane of the neoplastic cells. **c**-**f** Positive immunohistochemical staining for MUC1 (**c**) and MUC5AC (**d**) were observed, but CDX2 (**e**) and MUC2 (**f**) were negative. Original magnification: × 100 (**a**), × 200 (**b**-**f**)

A 68-year-old man on maintenance dialysis who was found to have a pancreatic mass on multidetector computed tomography (MDCT) taken for screening purposes was referred to our hospital. MDCT revealed a low-density mass with a diameter of 34 mm and no major vessel involvement in the tail of the pancreas, and multiple renal and liver cysts (e.g. polycystic kidney and liver diseases) (Fig. 1a). The levels of carbohydrate antigen 19–9 (CA19-9) and carcinoembryonic antigen (CEA) were significantly increased (13,077 U/ml and 62.7 ng/ml, respectively). Endoscopic ultrasound-guided fine needle aspiration was performed and the pancreatic tumor was pathologically diagnosed as adenocarcinoma. Further radiological examinations, including magnetic resonance imaging (MRI) of the brain (Fig. 1b), computed tomography (CT) of the chest (Fig. 1c), and positron emission tomography (PET) showed no evidence of distant metastasis. Considering these findings, the tumor was clinically diagnosed as resectable tail-PDAC, and the patient underwent distal pancreatosplenectomy with lymphadenectomy. Macroscopic examination showed white to tan solid mass with a diameter of 5.6 cm in the pancreatic tail (Fig. 2a, b). Histologically, neoplastic cells with predominantly clear formy cytoplasm infiltrate forming irregular shaped glands and nests, resulting in the diagnosis of moderately differentiated adenocarcinoma (Fig. 2c, d). Multifocal lymphovascular invasion was observed in the neoplastic tissue (Fig. 3a). Direct invasion to retropancreatic tissue was observed in some part, but surgical margin was free from the neoplastic cells. TNM classification based on the clinical, radiological and pathological findings was pT3, pN0, cM0 according to the 8^th^ edition of TNM classification by the American Joint Committee on Cancer/Union for International Cancer Control. Immunohistochemical staining revealed that neoplastic cells were positive for carbonic anhydrase 9 (CAIX), mucin core proteins 1 (MUC1) and MUC5AC, and negative for caudal-type homeobox 2 (CDX2) and MUC2 (Fig. 3b–f). The patient received gemcitabine as adjuvant chemotherapy for 6 months after surgery.

**Fig. 4:**
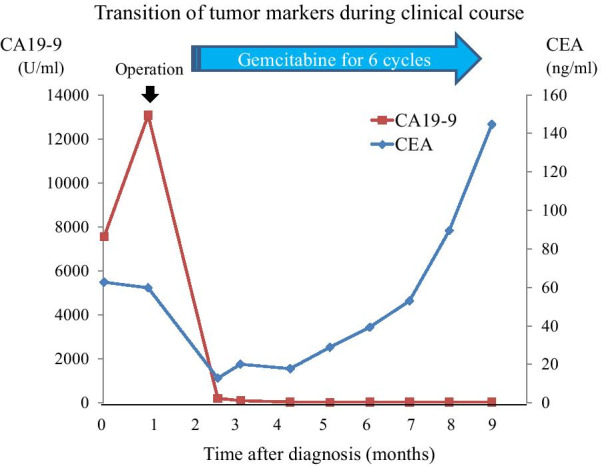
Transition of tumor markers during the clinical course. Changes in serum CEA and CA19-9 levels during the course of clinical treatment. CEA and CA19-9 levels were markedly reduced after surgery. Four months after the operation, the CEA level gradually increased, even in the course of adjuvant gemcitabine chemotherapy

**Fig. 5:**
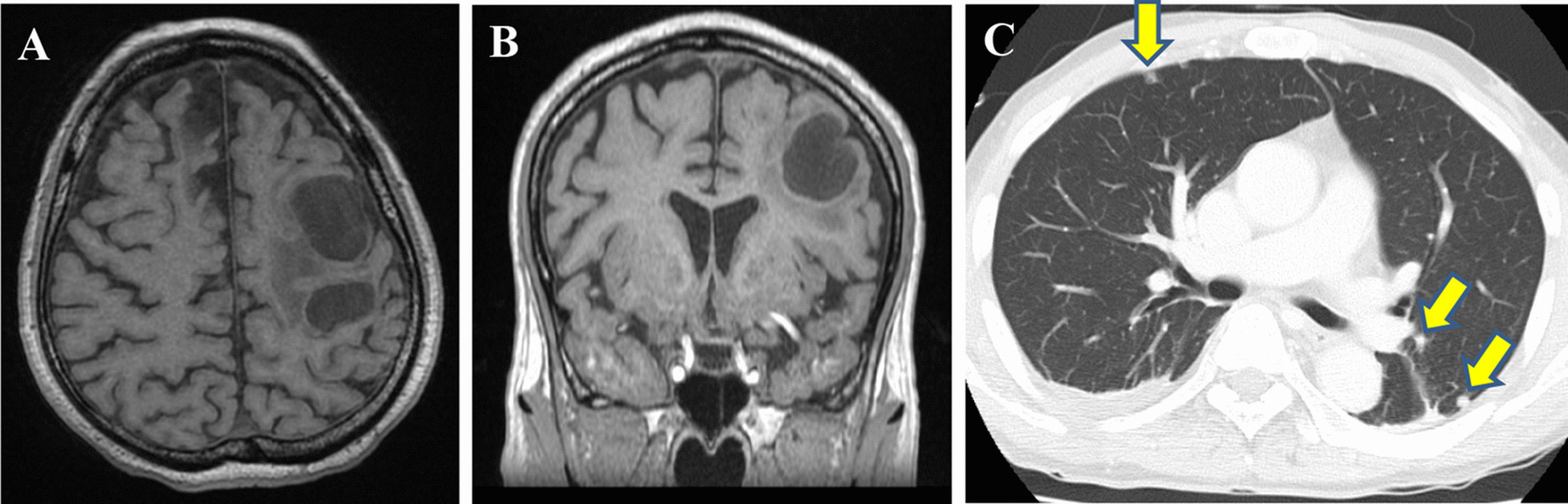
Radiological images of brain and lung metastases at 8 months after operation (**a**, **b**) Magnetic resonance imaging revealed brain metastasis at the left parietal lobe with two cystic lesions (**a**: axial view and **b**: coronal view). **c** Computed tomography revealed multiple metastases in both sides of the lungs (yellow arrows)

Eight months after surgery, serum CEA levels increased to 144.7 ng/ml (Fig. 4), and the patient presented to our hospital with right-sided weakness and dysarthria. MRI and CT showed distant metastases of PDAC in the brain and lung, simultaneously. Brain metastasis was located in the left parietal lobe as two cystic lesions (Fig. 5a, b), and multiple lung metastases were emerging on both sides of the lungs (Fig. 5c). He received gamma knife treatment for brain metastases. Subsequently, an intraventricular catheter (Ommaya reservoir) was inserted to drain the cystic fluid with turbid mucus. The cytology of the brain tumor indicated papillary atypical cells that were compatible with brain metastasis from the primary PDAC. Despite this treatment, the patient’s performance status gradually worsened and he received the best supportive care.

## Discussion

The first ante-mortem diagnosis of brain metastasis in a patient with PDAC was reported in 1977 [6]. To the best of our knowledge, only 28 cases in which brain metastases from PDAC were identified at the time of ante-mortem have been reported to date, including the present case (Table 1). The median age was 58 years (range 36–78 years), and the median time from diagnosis of PDAC to development of brain metastasis was 14 months (range 5–72 months). In our dataset, males were more frequently affected, representing 85% of patients, and 25 of all 28 cases (89.2%) had other metastatic sites such as liver (n = 12, 48%), lung (n = 8, 32%), bone (n = 3, 12%), or lymph node (n = 2, 8%). The most common site in PDAC with brain metastasis is the liver, which is consistent with that of overall PDAC metastasis after pancreatectomy [7–9]. Brain and lung metastases were identified simultaneously in the present case. The percentage of simultaneous brain and lung metastases was higher (32%) in the series of reviewed cohorts compared to that of PDAC patients who have recurrence in the lung after pancreatectomy (92 of 531, 17.3%) [7]. Indeed, Kumar et al. described that 3 of 8 (37.5%) PDAC patients with brain metastasis had lung metastasis in a large cohort study [9]. Recently, Sasaki et al. suggested that lung metastasis might be one of the risk factors for the development of brain metastasis in patients with PDAC as well as in patients with colorectal cancer [10, 11]. As a systemic disease, it can be inferred that cancer cells will develop brain metastasis via hematogenous dissemination beyond the blood–brain barrier, even if local recurrence is controlled. In 5 patients (17.8%), brain metastasis represented the first manifestation of PDAC, while the other patients developed brain metastasis later in the clinical course. It is speculated that brain metastases will be observed with increasing frequency due to the improved prognosis of PDAC patients.

Patient with polycystic kidney disease is famous in association with pancreatic cystic tumor as well as brain cystic tumor. We do not routinely perform a brain MRI in the initial staging. Specifically, in this case, the brain MRI was preoperatively performed because the patient was coincidentally detected an arachnoid cyst in his brain by preoperative PET-CT. Actually, there is a report that arachnoid cysts are an extra-renal manifestation associated with 8.1% of patients with autosomal dominant polycystic kidney disease [12]. Indeed, the metastatic brain tumor also showed cystic appearance. Taken together, it might be possible that there is a relation between cystic brain metastasis and polycystic kidney and liver disease in the present case. However, the primary pancreatic tumor did not show the cystic appearance and no cystic tumor was observed in pancreas of this case. Therefore, it seems to be no relevance between primary PDAC and his polycystic kidney and liver disease.

There are several options for the treatment of brain metastasis: surgical resection, whole-brain radiation therapy (RT), and stereotactic radiosurgery based on the European Association of Neuro-Oncology reported guidelines. In general, surgical resection should be considered in patients with a limited number [1–3] of newly diagnosed brain metastases [13]. Twelve of 28 patients underwent surgical resection with or without RT, but only three patients had survived for more than 6 years at the time their cases were reported. Hence, in patients for whom the primary tumor is uncontrolled, surgery for brain metastasis may not improve the prognosis. For the treatment of our patient, we selected gamma knife therapy and Ommaya reservoir placement to control the neurological symptoms, due to a decline in his activities of daily living and multiple lung metastases.

The characteristics of brain metastases in PDAC patients have been examined by IHC in order to understand the molecular basis of primary PDAC in some previous studies (Table 1). Consistent with our case, Chiang et al. reported that primary PDAC cells show positive staining for both MUC1, which is associated with the most invasive form of PDAC, and MUC5AC [14]. Further, PDAC activates KRAS and becomes hypoxic and dysplastic and refractory to chemotherapy and radiotherapy. To survive in a hypoxic environment, PDAC cells upregulate enzymes and transporters involved in pH regulation, including CAIX [15]. CAIX is upregulated in 34–78% of PDAC compared to normal pancreatic duct cells [16]. McDonald et al. demonstrated that hypoxia increases the resistance of human PDAC cells to chemotherapeutic agents, such as gemcitabine, and identified clinically tractable means of targeting CAIX, resulting in increased cell death with concomitant inhibition of tumor growth and dissemination [15]. Of particular interest, CAIX expression was substantially seen in primary PDAC cells in the present case. In our case, simultaneous metastases were observed in the brain and lungs just after adjuvant gemcitabine chemotherapy. No other such case has been reported to date. Considering these findings, it would be possible to influence CAIX-expressing PDAC cells’ resistance to gemcitabine; thus, the development of a CAIX inhibitor as an anti-metastatic agent for PDAC is expected in the future.

**Table 1:** Literature review of characteristics of PDAC patients with brain metastasis

First author ^ref^	yo, sex	Operation	Metastatic site	Period between BM Dx to PDAC Dx	Treatment to BM	OS from BM Dx	Molecular profiles
Kuratsu [17]	56yo, M 58yo, M	PD (-)	(-) Liver (S)	12 M 5 M	Ommaya + RT Res	Dead (9 M) Dead (2 W)	(-)
Chiang [18]	54yo, M	(-)	Liver (S)	S	Res + RT	Alive (> 20 M)	CK7( +), CK20(-) TTF-1(-), CDX2(-) MUC1/5AC( +), MUC2(-) KRAS^G12V^mutation
Caricato [19]	54yo, M	PD	(-)	24 M	Res	Alive (> 12 M)	(-)
Park [20]	48yo, M 51yo, M 52yo, M 62yo, M	(-)	Lung (S) Lung, Liver, Bone (S) Liver (S) Lung (S)	4 M S 5 M S	RT BSC RT BSC	MST 2.9 M (1.5 M-3.8 M)	(-)
El Kamar [4]	56yo, M	(-)	Liver (S)	6 M	chemoTX	Dead (3D)	CK7( +), CK20( +) TTF-1(-)
Lemke [21]	48yo, F 66yo, M	DP DP	Liver (36 M) (-)	72 M 12 M	Res + RT Res + RT	Alive (> 10Y) Alive (> 6Y)	(-)
Matsumura [22]	64yo, M	DP	LN (12 M)	14 M	Res + RT	Alive (> 10 M)	(-)
Marepaily [23]	36yo, F	(-)	Liver (S)	12 M	Res	Dead (< 1 M)	Adnab-9
Matsumoto [24]	68yo, M	(-)	Liver (S)	S	Res	Dead (3 M)	CK7( +), CK20(-)
Rajappa [25]	67yo, M	(-)	Liver (S), Lung (52 M)	48 M	Res + RT	Dead (36 M)	CK7( +), CK19( +) TTF-1(-)
Zaanan [26]	57yo, M	PD	Liver (6 M)	48 M	BSC	Dead (3D)	(-)
Rao [27]	58yo, M	(-)	Lung, Liver, Bone (S)	S	RT	Dead (< 3 M)	CK7( +), CEA( +) CK20/CDX2/ TTF-1(-)
Kumar [8]	Median 61.5yo (N = 8)	PD (n = 5) DP (n = 1) Partial (n = 1) (-) (n = 1)	Lung (n = 3) Liver (n = 2) Bone (n = 3) LN (n = 3)	Median period 29 M (2 M-57 M)	Reported (n = 4): Res + RT (n = 1) Res (n = 1) RT (n = 2)	> 9Y (post Res)	(-)
Matsuo [28]	61yo, F	(-)	Ascites (S)	16 M	Res	Dead (3 W)	(-)
Sasaki [9]	72yo, F 78yo, M	(-) DP	Liver (S) Lung (5 M)	19 M 28 M	RT RT	Dead (13 M) Dead (32D)	(-)
Our case	69yo, M	DP	Lung (8 M)	8 M	Ommaya + γknife	Alive (> 1 M)	CAIX( +) MUC1/5AC( +) CDX2/MUC2( +)

BM: Brain metastasis, BSC: best supportive care, D: days, DP: distal pancreatosplenectomy, Dx: diagnosis, LN: lymph node, M: months, MST: median survival time, N: no, Res: resection, OS: overall survival time, PD: pancreaticoduodenectomy, RT: radiotherapy, S: synchronous, Tx: therapy, Y: years, Y: Yes, yo: years old

In conclusion, we describe a case of simultaneous brain and lung metastases of PDAC after curative pancreatectomy. These data implicate that the metastatic niche between the brain and lung are similar, although the mechanisms for metastasis to both distant sites need further elucidation. The reported incidences of brain metastases will probably increase because the advances in treatment are leading to longer survival in PDAC patients. CAIX may be an important target for future treatment of PDAC brain metastasis.

## Data Availability

The datasets used and/or analyzed during the current study are available from the corresponding author on reasonable request.

## Abbreviations

CA19-9: Carbohydrate antigen 19–9
CAIX: Carbonic anhydrase 9
CDX2: Caudal-type homeobox 2
CEA: Carcinoembryonic antigen
MDCT: Multi-detector row computed tomography
MRI: Magnetic resonance imaging
MUC: Mucin core protein
PDAC: Pancreatic ductal adenocarcinoma
